# Draft Genome Sequence of the Ectomycorrhizal Ascomycete *Sphaerosporella brunnea*

**DOI:** 10.1128/MRA.00857-19

**Published:** 2019-12-12

**Authors:** Gian Maria Niccolò Benucci, Sajeet Haridas, Kurt Labutti, Giorgio Marozzi, Livio Antonielli, Sergio Sanchez, Pedro Marco, Xinxin Wang, Kerrie Barry, Anna Lipzen, Mansi Chovatia, Hope Hundley, Leonardo Baciarelli Falini, Claude Murat, Francis Martin, Emidio Albertini, Domizia Donnini, Igor V. Grigoriev, Gregory Bonito

**Affiliations:** a Department of Plant, Soil and Microbial Sciences, Michigan State University, East Lansing, Michigan, USA; b Great Lakes Bioenergy Research Center (GLBRC), East Lansing, Michigan, USA; c U.S. Department of Energy Joint Genome Institute, Walnut Creek, California, USA; d Department of Agricultural, Food and Environmental Science, University of Perugia, Perugia, Italy; e AIT Austrian Institute of Technology, Center for Health and Bioresources, Tulln, Austria; f Centro de Investigación y Tecnología Agroalimentaria de Aragón (CITA), Zaragoza, Spain; g UMR1136 Interactions Arbres-Microorganismes, Laboratoire d’Excellence ARBRE, INRA, Université de Lorraine, Champenoux, France; h Department of Plant and Microbial Biology, University of California Berkeley, Berkeley, California, USA; Vanderbilt University

## Abstract

Sphaerosporella brunnea is a pioneer ectomycorrhizal fungus with facultative saprophytic capacities. Here, we sequenced the genome of S. brunnea strain Sb_GMNB300, which is estimated at 51.6 Mb in size with 872 assembled contigs accounting for 12,597 predicted coding genes. This genome will be useful for comparative studies of Pezizales ectomycorrhizal symbioses.

## ANNOUNCEMENT

Sphaerosporella brunnea (Alb. & Schwein.) Svrček & Kubička is an ectomycorrhizal ascomycete in the Pyronemataceae family (Pezizales) that produces cup-shaped apothecia (1), often after rain or disturbance (2). S. brunnea grows in North America, Europe, Asia, and Oceania in association with trees and shrubs (3). It is a common cooccurring fungus of truffle-mycorrhized seedlings (2, 4, 5).

*Sphaerosporella brunnea* strain Sb_GMNB300 (NRRL 66913) was isolated from soil in Perugia, Italy, in April 2014. Mycelium from a single spore was grown in potato dextrose agar (PDA) medium for 10 days at 24°C. DNA was extracted with a modified cetyltrimethylammonium bromide (CTAB) protocol (6) and sequenced with PacBio technology using a low-input protocol for 10-kb libraries. The genome was assembled with Falcon v. 0.3.0 (7), improved with finisherSC v. 2.1 (8), and polished with Arrow SMRTLink v. 6.0.0.47841 (Pacific Biosciences, CA). Genome completeness was assessed using CEGMA v. 2.5 (9) and BBTools v. 38.38 (http://sourceforge.net/projects/bbmap). Contigs of less than 1,000 bp were excluded from the assembly. Mitochondrial reads were assembled separately. The genome was annotated using the Joint Genome Institute (JGI) annotation pipeline v. 2.0 (10, 11). The contents of the annotation pipeline are described in detail at https://mycocosm.jgi.doe.gov/programs/fungi/FungalGenomeAnnotationSOP.pdf.

For transcriptome analysis, mycelium was grown in PDA and malt extract agar (MEA) media at 24°C and 4°C for 10 and 20 days, respectively. RNA was extracted with the RNeasy plant minikit (Qiagen, Germany), and the library was prepared with an Illumina TruSeq stranded mRNA HT sample prep kit with poly(A) selection of mRNA and sequenced using a NovaSeq 6000 2 × 150-bp sequencer (Illumina, CA). After sequencing, read artifacts (kmer = 25 bp, 1 mismatch) were detected with BBDuk v. 38.34 (https://sourceforge.net/projects/bbmap/). Detected artifacts were trimmed from the 3′ end of reads. General quality trimming was performed with the Phred trimming method set at Q6. Reads shorter than 25 bp or one-third of the original read length were removed, as well as RNA spike-in reads, PhiX reads, and reads containing any ambiguous characters (Ns). Clean transcriptome sequencing (RNA-Seq) reads were assembled in Trinity v. 2.3.2 (12). RNA-Seq capture was performed by aligning a 1% subsample of RNA reads to the final DNA assembly using BBTools v. 38.67. Gene clusters of interest were searched in the clustering table available at the JGI MycoCosm portal (https://mycocosm.jgi.doe.gov/clm/run/Sphbr2-comparative-qc.3266?organism=Sphbr2). Clusters were computed following the TRIBE-Markov cluster (MCL) clustering method of Enright et al. (13), from all-versus-all BLAST analysis of the proteins in the set of organisms included in a clustering run.

The *Sphaerosporella brunnea* draft genome had an estimated size of 51,598,955 bp, sequence coverage depth of 907.91×, 872 (≥2 kbp) total contigs (and scaffolds), an *N*_50_ value (i.e., the smallest number of contigs whose length sum makes up half of the genome size) of 130, an *L*_50_ value (i.e., half of the genome sequence is in contigs larger than or equal to this length) of 119,241 bp, and an estimated average GC content of 52.64% ± 2.33%. The mitochondrion genome is in 5 contigs and is 265,525 bp in size (*N*_50_ = 2, *L*_50_ = 78,073 bp). A Core Eukaryotic Genes Mapping Approach (CEGMA) completeness score of 99.34% and an RNA-Seq capture of 96.85% were obtained for the final assembly.

The *Sphaerosporella brunnea* genome size is similar to those of other Pyronemataceae species (e.g., Pyronema confluens [50.0 Mbp] [14]) but smaller than those of Tuberaceae species (e.g., Tuber aestivum [145.0 Mbp] [15], Tuber melanosporum [125.0 Mbp] [16], and Tuber borchii [97.2 Mbp] [17]). A total of 12,597 protein-coding genes were detected, with average lengths of 1,555 bp and 397 amino acids for genes and proteins, respectively. The number of genes is similar to those of P. confluens (13,367) and T. borchii (12,346) but greater than those of T. melanosporum (10,058) and T. aestivum (9,344). Predicted proteins in the genome of *S. brunnea* demonstrate an enriched capacity to produce plant cell wall-degrading enzymes compared to that of its relatives, which includes 2 genes in glycoside hydrolase family 6 (GH6) compared to 1 present in *P. confluens* and 3 genes in GH7 compared to 2 in *P. confluens* that are absent in Tuber spp. (16). Only the mating type locus MAT1-2-1 with the high-mobility-group (HMG) box (protein identifier 924729, https://mycocosm.jgi.doe.gov/cgi-bin/dispGeneModel?db=Sphbr2&id=924729) was identified in the genome of *S. brunnea* Sb_GMNB300, in contrast to reports by Sánchez and colleagues (3) and expectations of homothallism.

### Data availability.

This whole-genome shotgun project has been deposited at DDBJ/ENA/GenBank under the accession number VXIS00000000. The version described in this paper is the first version, VXIS01000000. Genome and transcriptome data are available in DDBJ/ENA/GenBank under BioProject number PRJNA554466, BioSample number SAMN12268380, and Sequence Read Archive accession numbers SRP214822 and SRP215074, or on the U.S. Department of Energy (DOE) JGI MycoCosm portal (9) (comparative).
